# Does experience matter? Understanding the mechanism of the volume-outcome relationship: Learning-by-doing or economies of scale

**DOI:** 10.1371/journal.pone.0318808

**Published:** 2025-03-26

**Authors:** Ritesh Maharaj

**Affiliations:** Department of Health Policy, London School of Economics and Political Science, London, United Kingdom; Universiti Sains Malaysia, MALAYSIA

## Abstract

**Objective:**

To evaluate the underlying mechanism of the volume-outcome relationship, namely learning-by-doing and scale economies in patients with sepsis.

**Design and study setting:**

Retrospective cohort study of adult patients with sepsis between 1 January 2010 and 31 December 2016 in 231 intensive care units (ICUs) in the UK.

**Participants:**

The patient was the primary unit of analysis. Patient and ICU characteristics were included for risk adjustment. Demographic and clinical data were extracted from the Intensive Care National Audit and Research Centre (ICNARC) Case Mix Programme database.

**Study design:**

We used the lags of quarterly sepsis volume in the ICU as a measure of the learning-by-doing effect.

**Outcome measure:**

The outcome of hospital mortality after ICU admission for sepsis was assessed using a multilevel probit regression model of patients nested in ICUs over quarters.

**Data collection/extraction methods:**

Critically ill patients with sepsis were identified by the Sepsis-3 consensus criteria.

**Results:**

Our study identified a cohort of 273001 patients with sepsis admitted to 231 ICUs in the UK. Our study finds that in comparison with contemporaneous volume, lagged volume had a stronger association with acute hospital mortality. This implies that the dynamic learning-by-doing effect is more important than the static economies of scale effect. This finding was consistent across alternate specifications of learning-by-doing.

**Conclusions:**

The study provides evidence that the underlying mechanism for the volume-outcome relationship is learning-by-doing and not the static economies of scale. ICUs caring for patients with sepsis tend to improve by experience.

## Introduction

The volume-outcome relationship has been a commonly invoked policy initiative aimed at improving the quality of healthcare. This inverse relationship between the caseload volume of patients treated and patient mortality has been described across many health settings and in many countries [1–3]. Despite the large body of literature demonstrating this favourable relationship, most studies have focused on differentiating the effects of selective referral and the true effects of volume. In comparison, there are few studies evaluating the underlying mechanism of the volume-outcome relationship, namely dynamic learning-by-doing or the static effect of economics of scale [3,4]. Resolving this tension between policies that allow providers to accrue experience over time and policies that promote centralisation of services would make a more compelling argument for policies such as minimum volume standards to be firmly established [5–10].

### Conceptual framing of dynamic learning-by-doing versus the static scale effect

The term learning-by-doing was introduced by Arrow in 1962 to refer to institutional learning and describes the improvements in outcomes by experience [11]. The basic idea is that the team acquires experience by performing tasks repeatedly and that care improves because of the cumulative effect of skills gained by patients treated in the past. The knowledge is gained through the production of experience as activity increases and has been termed a learning-curve [11]. Learning-by-doing would result in increases in quality attributed to increases in knowledge. The acquisition of knowledge is a product of experience [11]. Even when volume remains unchanged between time periods, improvements in quality attributable to experience still occur. Learning takes place through activity and can be observed as improving performance so that today’s volume improves tomorrow’s outcomes.

**Fig 1:**
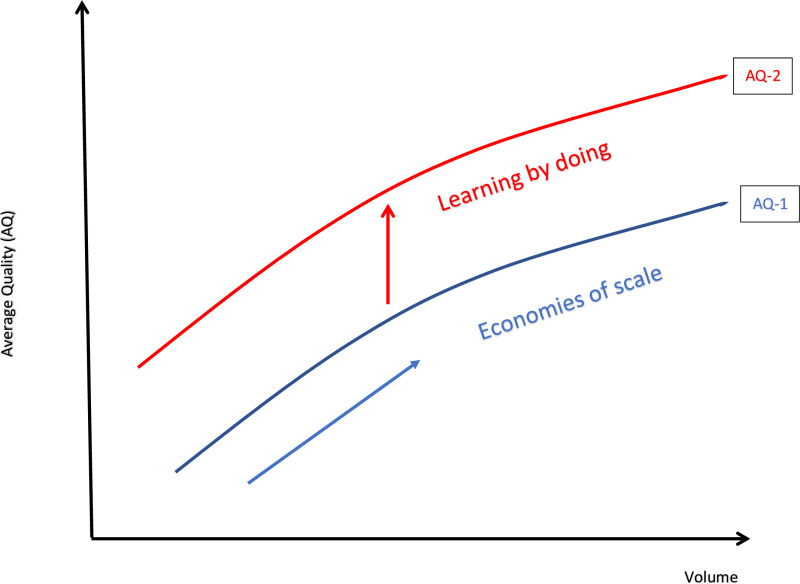
Economies of scale versus learning-by-doing. The blue arrow refers to movement along the Average Quality (AQ 1) curve reflecting the effect of economies of scale. The red arrow refers to changes in productivity by experience or learning by doing, resulting in a new Average Quality curve AQ-2. The distinction between static economies of scale and dynamic learning-by-doing is important because if the volume-outcome operated entirely through movement along the economies of scale curve (AQ-1), such as by investments in infrastructure and research and development, then equating static marginal quality to marginal volume would be socially optimal. Consider the example of a transitory shock that raises short-term demand such as a pandemic, assuming demand does not exceed a supply threshold. In such a scenario the economies of scale mechanism predicts no long-run gains to quality when volume returns to baseline. In contrast, the learning-by-doing mechanism would predict a permanent.

The alternate mechanism of the volume-outcome relationship is the static scale effect. Here economies of scale are driven by indivisibilities of critical investments which impact patients’ outcomes and reflect contemporaneous gains provided through consolidation or centralisation. In the economies of scale mechanism, today’s caseload volume affects contemporaneous outcomes. The distinction between these mechanisms is described in Fig 1.

From a policy perspective, the underlying mechanism by which volume leads to positive patient outcomes matters. If the static volume is the underlying mechanism, then any ICU in which volume is concentrated will lead to better outcomes and centralizing care in a small number of large ICUs will lead to better patient outcomes. Alternatively, if experience is the underlying mechanism, then shifting volume from one ICU to another would reduce opportunities for learning in the ICU transferring patients out. This would make ICU consolidation a less beneficial policy.

The evidence supports two key mechanisms in the volume-outcome relationship: static economies of scale and learning-by-doing. Their prominence appears to vary depending on the type of patient cohort under study [9,11]. Existing literature has focused largely on coronary revascularization and elective surgeries, while non-surgical contexts remain underexplored. Most studies use mortality as an outcome to investigate the volume-outcome relationship and its underlying mechanisms. Studies of elective surgeries with low mortality rates tend to support static economies of scale, whereas research on more complex surgeries with higher mortality often attributes improvements to learning-by-doing. For example, in heart transplantation, successive cost reductions suggest that performance improvements are driven by accumulated experience rather than economies of scale [12]. Gaynor et al. examined the effects of scale economies and learning-by-doing in cardiac surgery, concluding that the benefits of volume were primarily due to static scale economies [9]. A major limitation of this work was the year lag structure used to detect a learning-by-doing effect meant that learning over shorter periods was not observable. Similarly, Ho et al. examined coronary angioplasty and found better outcomes with higher annual hospital volumes but no evidence of learning-by-doing [13]. Another U.S.-based study on cardiac procedures also found no learning effects, attributing all volume-related benefits to static scale economies [14]. A common critique of the literature lies in its reliance on cumulative volume for procedures like coronary angioplasty, a treatment already well-established in clinical practice. This approach makes it unclear when practitioners first gained exposure to the procedure, complicating efforts to isolate the effects of experience on outcomes [15]. Another critique is the use of mortality as an outcome for elective cardiac procedures when it is an uncommon event. In cohorts where mortality is more common, such as advanced cancer surgery patients, a substantial learning-by-doing effect has been identified, with lagged volume showing a stronger impact than contemporaneous volume [16]. This effect was particularly pronounced in patients with colon cancer compared to those with breast cancer, suggesting that experience provides greater benefits when managing diseases of higher complexity.

The two major limitations of the current literature on the mechanism of the volume-outcome relationship that we aim to address in this study can be summarised as follows [4]. First, almost all the evidence of the mechanism of the volume-outcome relationship is derived from elective surgical populations and predominantly cardiac and cancer-related surgery. In these surgical populations, there may be selective referral to higher-performing centres. In contrast to elective surgery, patients with sepsis are usually taken to their nearest ICU, removing selection related to ICU quality or severity of illness. Second, all of the previous studies use a fixed-effects approach to account for time-invariant institution-level unobserved heterogeneity [17]. The inclusion of an institutional fixed-effect means that the regression estimates the effects of changes to volume within the hospital rather than the effects of changes to cumulative volume across hospitals on mortality [17]. The fixed-effects specification requires an adequate number of institutions with significant variation in volume to detect a learning-by-doing effect. Previous studies that have failed to show a learning-by-doing effect may not have been suitably powered [17].

Sepsis patients are the ideal cohort to study the underlying mechanism of the volume-outcome relationship. Sepsis is a major public health concern and the leading cause of morbidity and mortality globally with 1 in 5 deaths worldwide attributed to sepsis [18]. The Seventeenth World Health Assembly recognised the importance of strong, functional health systems including access to intensive care services and health system organizational strategies to improve outcomes from sepsis [19]. One strategy to improve outcomes has been centralisation of care [7,20]. Centralisation is predicated on the assumption that the volume-outcome relationship operates through the static scale effect. If the volume-outcome relationship operates through the learning-by-doing mechanism, then patient outcomes would improve by experience, making system-wide centralisation unnecessary. The unsettled question of the underlying learning mechanism in the volume-outcome relationship therefore has clear implications to all stakeholders in the health system.

We make several contributions to the literature on the volume-outcome relationship. First, this study measures volume at a higher frequency than previously done (quarter instead of year). Given the temporal instability of ICU teams and of hospital teams generally, it is likely that learning might occur over shorter time periods and then undergo some decay as teams change. The quarterly time lags are more likely to detect learning over shorter periods than year lags. Second, our study includes many ICUs over several years and employs a mixed-effects probit regression model. This approach is more sensitive to detecting a learning-by-doing effect than previous fixed-effects approaches. Precise estimation using the fixed-effect approach requires data from enough institutions over a significant number of time periods to observe sufficient variation in volume. Previous studies have contained small sample sizes in terms of number of institutions which may therefore be underpowered to detect a learning-by-doing effect [17]. Third, we control for a rich set of patient and ICU characteristics to minimise the risk of omitted variable bias. Lastly, this paper ties the less commonly reported literature on the underlying mechanism of the volume-outcome relationship, namely economies of scale and learning-by-doing, with mortality and provides useful information on how reconfiguring service lines may improve underperforming lower volume ICUs.

## Methods

### Data

Data was extracted from the Intensive Care National Audit and Research Centre Case Mix Program database which is a clinical database that covers all adult ICUs in England, Wales, Northern Ireland, United Kingdom [21]. Trained data collectors extracted detailed physiological, diagnostic, and sociodemographic data from consecutive adults admitted to ICUs in the United Kingdom participating in the Case-Mix Program database between 1 January 2010 and 31 December 2016 [22]. Approval for the collection and use of patient identifiable data in the CMP was obtained under Section 251 of the National Health Service Act of 2006. The London School of Economics waived the requirement for approval and informed consent because this research involved secondary analysis of an established dataset of anonymised data. We report an observational cohort study, as per Strengthening the Reporting of Observational Studies in Epidemiology (STROBE) guidelines [23].

### Exposure

The exposure was defined as by the quarterly sepsis volume with contemporaneous quarterly volume being the measure of the static scale effects and the lagged quarterly volume identifying the learning-by-doing effect.

### Study outcome

The primary outcome was death before discharge from an acute hospital. Patients who were transferred between ICUs were excluded from the analysis of mortality but included in the estimation of ICU volumes. This was done to avoid confounding results with outcomes from different ICUs. For patients who were readmitted to the ICU, only the first admission was included in the mortality analysis. We chose the unit of analysis to patient mortality. An ICU-level analysis of sepsis volume and mortality would smooth out variability in outcomes across patients [13]. In the case of no observed learning effect, it would be unclear as to whether this is due to data aggregation or a true absence of a learning effect.

### Empirical strategy

Baseline characteristics and unadjusted outcomes for the cohort were tabulated using standard summary statistics. We used a multivariate hierarchical probit regression model to assess the association between volume and acute hospital mortality. Our model recognises that individual patients clustered in quarters and nested in ICUs and provides a consistent estimate of the standard errors for clustered data.

Patient-level covariates include age, gender, ethnicity, functional status, co-morbidities, and sociodemographic status as measured by the Index of Multiple Deprivation. We measure patient level severity of illness using the ICNARC_2018_ score. We include dummy variables for the presence of severe co-morbidities involving 7 organs systems. Functional status was categorised by the degree of assistance needed with activities of daily living. ICU characteristics are quarterly caseload volume, academic affiliation (non-university, university, university-affiliated) and quarterly throughput. Quarterly throughput is defined as the number of ICU admissions per ICU bed.

In the first step we will focus on overall learning curves in hospital mortality. We describe the basic model of Benkard with the important difference that the model presented here will involve multiple ICUs where the initial experience is unknown [24]. The simplest specification of the volume-outcome relationship is:


$$y_{ijq}=\beta_{1}V_{jq}+\beta_{2}V_{jq-1}+\overset{M}{\underset{m=1}{\sum }}\delta_{m}X_{ijq}^{'}+\overset{N}{\underset{n=1}{\sum }}\phi_{n}Z_{jq}^{'}+\xi_{j}+\varepsilon_{ijq}$$

The dependant variable $y_{ijq}$ is patient-level mortality of patient *i* in ICU jin quarter q. $X_{ijq}^{'}$ is a vector of patient characteristics and $Z_{jq}^{'}$ is a vector for ICU level characteristics. $V_{jq}$ refers to quarterly ICU sepsis volume and captures the effects of static scale. The coefficient of $V_{jq-1}$ is the sepsis volume in e preceding quarter and describes the ICU-level learning-by-doing effect. The $\xi_{jq}$ captures to the ICU effect and $\varepsilon_{ijq}$ is a classical error term.

We can expand the learning-by-doing component by including four lags of sepsis volume.


$$y_{ijq}=\beta_{1}V_{jq}+\overset{q-4}{\underset{q^{\prime }=q-1}{\sum }}\beta_{q^{\prime }}V_{jq^{\prime }}+\overset{M}{\underset{m=1}{\sum }}\delta_{m}X_{ijq}^{'}+\overset{N}{\underset{n=1}{\sum }}\phi_{n}Z_{jq}^{'}+\xi_{j}+\varepsilon_{ijq}$$

The individual weights of $\beta_{q^{\prime }}$ are called lag weights and they collectively constitute the lag distribution from $q^{\prime }=q-1\ldots q-4$, with the full set of quarters being $q=\left\{q,q^{'}\right\}.$ The lags in volume estimate the effects of learning over time. If there was learning-by-doing and knowledge was passed on from one period to the next, we would observe a larger coefficient with each succeeding time period, i.e., $\beta_{q-1}>\beta_{q-2}>\beta_{q-3}>\beta_{q-4}$. This is because learning-by-doing allows patients treated in the current time period to benefit from experience gained in the preceding time periods.

The ICU effect in the quarter q−1 makes it unnecessary to know the ICU’s entire production history. We separate out the volume-outcome effects into its static and dynamic components. Instead of using cumulative learning treating all past periods as the same, we use lags of the previous quarters’ volumes of sepsis. We compare the relative size of the coefficients. If the static scale economies is the main mechanism for the volume outcome relationship, then the coefficients of the lagged volumes would be small, i.e., ($\beta_{1}>\beta_{q-1}+\beta_{q-2}+\beta_{q-3}+\beta_{q-4}).$If the learning-by-going is important then the coefficients on the lagged volume would be a larger proportion of the total effect. This would imply that experience gained in the past impacts the outcomes of the present. If the contemporaneous volume accounts for a larger proportion of the effect, then it would mean there are benefits to static scale. This would imply that any ICU high volume ICU would improve outcomes and that there would be benefits to indivisibilities of investments in infrastructure favouring consolidation of critical care services.

Quarterly volumes are correlated over time, meaning that $V_{jq}$ is correlated with $V_{jq-1}$, as are $V_{jq-1}$and $V_{jq-2}$correlated as well as $V_{jq-2}$and $V_{jq-3}$. High level correlation between regressors, referred to as multicollinearity, leads to unreliable coefficient estimates with large variances and standard errors. This leads to lag distributions in which the sequence of lag coefficients bounces between large and small and even sometimes positive and negative. We describe the distribution of the correlation coefficients between the volume lags. A weaker correlation between volume lags would support a low risk of multicollinearity.

### Sensitivity analysis

The main variable of interest is in capturing the learning mechanism. In the primary analysis we used quarterly lags to identify a linear learning-by-doing-effect. We undertook two sensitivity analyses to identify other specifications of the learning-by-doing mechanism. First, we used monthly sepsis volume to detect any learning that may occur over shorter time periods. Second, we specify quarterly volume a simple square root form to identify a non-linear learning-by-doing relationship.

## Results

The study population of adult sepsis patients admitted to the ICU was 273,001. The median quarterly sepsis volume was 63 IQR [46–86]. Table 1 summarises the patient characterises across quartiles of quarterly caseload volumes from 2010 to 2016. The mean age of patients was 63 (95% CI 63-63) years. A minority of patients had a severe comorbidity (20.3%) and most patients were functionally independent (68.0%). The mean ICNARC_2018_ predicted risk of acute hospital mortality was 21.0% (95%CI 21.0b -21.0). Across quartiles of sepsis caseload volume, patients treated in the lowest quartile had higher acute severity of illness scores ICNARC score 21.3 [21.2-21.4] compared with 20.5 [20.5-20.6] in the highest quartile, p < 0.001. Lower sepsis volume ICUs treated more patients with no chronic comorbidities compared with higher volume ICUs where 81.3% in the lowest quartile had no comorbidities compared with 76.3% in the highest quartile. On average, higher volume ICUs operate at higher occupancy and have higher throughput of patients. The trend in monthly, quarterly and six-monthly volumes from 2010 to 2016 and the mean lagged volumes are described in S1 Table in the supporting information.

**Table 1:** Patient characteristics across quartiles of quarterly sepsis volume.

	Total	Quartile I [4-46]	Quartile II [47-63]	Quartile III [64-86]	Quartile IV [87-226]	P value
**Age in years n(%)**						
≤*53*	68357 (25.0)	17490 (24.4)	16787 (24.4)	16785 (25.3)	17295 (26.4)	<0.001
*54-66*	68460 (25.1)	17957 (25.0)	16947 (24.6)	16702 (25.2)	16854 (25.6)	
*67-76*	70835 (25.9)	18890 (26.3)	18016 (26.2)	17348 (26.1)	15809 (25.1)	
≥*77*	65339 (23.9)	17452 (24.3)	17094 (24.8)	15573 (23.5)	15520 (23.0)	
**Age in years Median [IQR]**	63 [63-63]	64 [64-64]	64 [64-64]	63 [63-63]	63 [63-63]	<0.001
**Male sex n(%)**	148149 (54.3)	38734 (54.0)	37114 (53.9)	36287 (54.7)	36014 (54.6)	0.004
**Ethnicity n(%)**						
*White*	277787 (92.3)	73742 (92.3)	69996 (92.2)	68379 (92.1)	65670 (86.8)	<0.001
*Asian*	10723 (3.5)	2682 (3.4)	2225 (2.9)	2310 (3.1)	3506 (4.6)	
*Black*	6192 (2.0)	1328 (1.7)	1353 (1.8)	1066 (1.4)	2445 (3.2)	
*Mixed/other*	10855 (3.5)	2178 (2.7)	2261 (3.0)	2375 (3.2)	4041 (5.3)	
**ADLs**						
*Independent*	184850 (68.0)	49229 (68.6)	46876 (68.1)	43940 (66.2)	44805 (67.9)	<0.001
*Some assistance*	81913 (30.1)	20927 (29.2)	20416 (29.7)	20985 (31.6)	19585 (29.7)	
*Fully dependent*	5071 (1.9)	1325 (1.9)	1252 (1.8)	1187 (1.8)	1307 (2.0)	
**ICNARC score mean (95% CI)**	21.0 [21.0-21.0]	21.3 [21.2-21.4]	21.1 [21.0-21.2]	21.0 [20.9-21.0]	20.5 [20.5-20.6]	<0.001
**APACHE II, mean (95% CI)**	18.4 [18.4-18.5]	18.4 [18.4-18.5]	18.3 [18.3-18.4]	18.4 [18.4-18.5]	18.6 [18.6-18.7]	<0.001
**Occupancy %, (95% CI)**	72 [72-73]	68 [68-68]]	72 [72-72]	74 [74-74]	77 [77-77]	<0.001
**ICU beds mean, (95% CI)**	15 [14,14]	9 [9,9]	12 [–]	16 [15,15]	24 [23,23]	<0.001
**Quarterly throughput**	5.0 [5.0-5.0]	4.3 [4.3-4.3]	5.1 [5.1-5.1]	5.2 [5.2-5.2]	5.3 [5.3-5.3]	<0.001
**Co-morbidities n(%)**						
*None*	217655 (79.7))	58354 (81.3)	55821 (81.1)	53,156 (80.0)	50324 (76.3)	<0.001
*Cardiac disease*	4857 (1.8)	1398 (2.0)	1151 (1.7)	1243 (1.9)	1065 (1.6)	<0.001
*Respiratory disease*	12498 (4.6)	3100 (4.3)	3002 (4.3)	2923 (4.4)	3473 (5.3)	
*ESKD*	5171 [1.9)	953 (1.3)	1075 (1.6)	1230 (1.9)	1913 (2.9)	<0.001
*Liver disease*	6030 (2.2)	1213 (1.7)	1315 (1.9)	1428 (2.2)	2074 (3.1)	<0.001
*Metastatic cancer*	6598 (2.4)	1677 (2.3)	1529 (2.2)	1620 (2.4)	1772 (2.7)	<0.001
*Hematologic malignancy*	9763 (3.6)	2377 (3.3)	2235 (3.3)	2341 (3.5)	2810 (4.3)	<0.001
*Immunocompromised*	24035 (8.8)	6012 (8.4)	5706 (8.3)	5803 (8.7)	6514 (9.9)	<0.001
Septic shock	54419 (19.9)	14961 (20.8)	13907 (20.2)	12703 (19.1)	12848 (19.5)	<0.001
ICU LOS (hrs)Median[IQR]	163 [162-164]	169 [168-171]	159 [157-160]	163 [162-165]	159 [157-161]	<0.001
Hospital LOS (days) Median[IQR]	23 [22,22]	23 [22,22]	22 [22-22z]	23 [22,22]	24 [23,23]	<0.001
ICU mortality n(%)	62277 (22.8)	16868 (23.5)	15919 (23.1)	15196 (22.9)	14294 (21.7)	<0.001
Hospital Mortality n(%)	86728 (31.8)	23650 (32.9)	21944 (31.9)	20932 (21.6)	20202 (30.6)	<0.001

Abbreviations: IQR=interquartile range, ADLs = Activities of daily living, ESKD =  end-stage kidney disease.

Data were missing for ADLs 1,167 (0.4), comorbidities 1137 (0.4%); ICU mortality 7 (0.0%); hospital mortality 1419 (0.5%).

The distribution of the correlation coefficients between successive lags is described by the violin plots in S1 Fig in the Supporting information. The median correlation between successive lagged quarterly sepsis volumes ranged from 0.255 [IQR 0.056-0.467], to 0.267 [IQR 0.091-0.482]. These values suggest a weak to moderate correlation between lagged volumes and low risk of multicollinearity.

In Tables 2 and 3, we present the marginal effects and coefficients of the contemporaneous and lagged effects of volume on hospital mortality. We show that the lagged effect is significant in each of the models. The sum of the total effect represented by the F-test is more reliably estimated across models. The F-test for the combined effect of the lagged volume remains significant. The test of joint significance returned a p-value of 0.005, < 0.001, < 0.001 and 0.007 for the 1,2,3 and 4^th^ quarterly lag, respectively. In Table 3, we further explore the relative relationship between the contemporaneous and lagged volume. The ratio of the coefficient for the contemporaneous volume to the sum of all the volume coefficients was used to describe the relative importance of static scale economies. The contemporaneous volume accounts for 21%, 34%, 53% and 28% of the total effect of volume, for the 1,2,3, and 4^th^ lag respectively. The results suggest that the contemporaneous volume did not constitute the dominant mechanism for the volume outcome relationship. The ratio of successive lagged coefficients was used to identify the strength of the learning curve. A ratio of greater than 1 would suggest complete knowledge retention. The ratio between the first and second quarter was low, ranging from 3% to 14%. The ratio between the second and third quarter was 166% to 163% and the third and fourth quarters was 193%. This would suggest that learning takes two to three quarters with some degradation of learning.

**Table 2:** Probit regression results.

Covariates	(1)	(2)	(3)	(4)	(5)
$Volume_{q}$	−0.00024***	−0.00005	0.00009	0.00015	0.00006
	(0.0001)	(0.0001)	(0.0001)	(0.0001)	(0.0001)
$Volume_{q-1}$		−0.00020**	−0.00004	−0.00003	−5.82$e^{-6}$
		(0.00007)	(0.0001)	(0.0001)	(0.0001)
$Volume_{q-2}$			−0.00033***	−0.00024**	−0.00021**
			(0.0001)	(0.0001)	(0.0001)
$Volume_{q-3}$				−0.00015*	−0.00013
				(0.0001)	(0.0001)
$Volume_{q-4}$					0.00007
					(0.0001)
F-statistic		8.51**	21.19***	22.02***	7.33***
**Patient characteristics**					
*ADLs*					
Independent	*Reference*	*Reference*	*Reference*	*Reference*	*Reference*
Some assistance	0.01233***	0.01191***	0.01197***	0.01153***	0.01153***
	(0.0018)	(0.0018)	(0.0019)	(0.0019)	(0.0019)
Total assistance	0.04594***	0.04348***	0.04461***	0.04370***	0.04394***
	(0.0061)	(0.0062)	(0.0064)	(0.0066)	(0.0067)
*Ethnicity*					
White	*Reference*	*Reference*	*Reference*	*Reference*	*Reference*
Asian	−0.00133	−0.00129	−0.00059	−0.00142	−0.00070
	(0.0043)	(0.0044)	(0.0045)	(0.0047)	(0.0048)
Black	−0.02175***	−0.02175***	0.02283***	0.02416***	0.02424***
	(0.0056)	(0.0053)	(0.0061)	(0.0062)	(0.0064)
Mixed/other	−0.00159	−0.00159	−0.00155	−0.00339	−0.00416
	(0.0043)	(0.0043)	(0.0046)	(0.0048)	(0.0049)
Age in years	0.00168***	0.00167***	0.00172***	0.00170***	0.00172***
	(0.0000)	(0.0000)	(0.0001)	(0.0001)	(0.0001)
Male	0.01459***	0.01459***	0.01549***	0.01580***	0.01564 ***
	(0.0015)	(0.0015)	(0.0016)	(0.0016)	(0.0016)
ICNARC score	0.00704***	0.00704***	0.00678***	0.00678***	0.00678***
	(0.0000)	(0.0000)	(0.0000)	(0.0000)	(0.0003)
*Comorbidity*					
Severe respiratory disease	0.00838*	0.00921*	0.00875*	0.00927*	0.00935*
	(0.0035)	(0.0036)	(0.0036)	(0.0037)	(0.0038)
Very severe cardiovascular	0.04459***	0.04504***	0.04443***	0.04534***	0.04672***
	(0.0053)	(0.0054)	(0.0055)	(0.0056)	(0.0058)
ESKD	0.04328***	0.04151***	0.03935***	0.03952***	0.04066***
	(0.0051)	(0.0052)	(0.0054)	(0.0055)	(0.0056)
Severe liver disease	0.06082***	0.06038***	0.06109***	0.06044***	0.05971***
	(0.0048)	(0.0049)	(0.0050)	(0.0051)	(0.0052)
Metastatic disease	0.02381***	0.02381***	0.02523***	0.02359***	0.02171***
	(0.0047)	(0.0047)	(0.0048)	(0.0049)	(0.0051)
Haematological malignancy	0.01464***	0.01384***	0.01570***	0.01501***	0.01393**
	(0.0041)	(0.0041)	(0.0042)	(0.0043)	(0.0044)
Immunocompromised	0.02191***	0.02191***	0.01935***	0.01994***	0.019923***
	(0.0028)	(0.0029)	(0.0029)	(0.0030)	(0.0031)
IMD^@^	−7.00$e^{-7}$***	−7.13$e^{-7}$***	−7.37$e^{-7}$***	−7.38$e^{-7}$***	−7.40$e^{-7}$***
	(8.96$e^{-8}$)	(9.14$e^{-8}$)	(9.43$e^{-8}$)	(9.64$e^{-8}$)	(9.90$e^{-8}$)
**ICU characteristics**					
Quarterly throughput	0.00298***	0.00196***	0.00053	0.00036	0.00092
	(0.0009)	(0.0009)	(0.0010)	(0.0011)	(0.0011)
*Academic affiliation*					
Non-university	*Reference*	*Reference*	*Reference*	*Reference*	*Reference*
University affiliated	−0.00310	−0.00307	−0.00285	−0.00255	−0.00327
	(0.0060)	(0.0053)	(0.0062)	(0.0062)	(0.0063)
University	0.00640	0.00717	0.00722	0.00688	0.00711
	(0.0052)	(0.0053)	(0.0055)	(0.0056)	(0.0057)
AIC	231765.08	220498.8	210757.9	201056.3	189384.6
BIC	232005.71	220748.8	211017.1	201324.7	189661.8
F-statistic		8.51**	21.19***	22.02***	7.33***

@ = IMD = Index of Multiple Deprivation.

P < 0.05 = *, P < 0.01 = **, P < 0.001 = ***.

**Table 3:** Probit regression model showing coefficients, the proportion of the contemporaneous volume to the total volume efcts and the ratio of lagged coefficients.

	Models				
Lag depth	(1)	(2)	(3)	(4)	(5)
0	−0.00024	−0.00021	0.00065	0.00106	0.00044
1		−0.00079	−0.00028	−0.00025	−0.00004
2			−0.00231	0.00174	−0.01500
3				−0.00105	−0.00921
4					0.00048
**Total**	−0.00024	−0.00100	−0.00194	−0.00198	−0.00155
$\frac{\beta_{1}}{\sum_{n=0}^{\infty }\beta_{n}}$	1.00	0.21	0.34	0.53	0.28
$\frac{\beta_{2}}{\beta_{3}}$			0.12	0.14	0.03
$\frac{\beta_{3}}{\beta_{4}}$				1.66	1.63
$\frac{\beta_{4}}{\beta_{5}}$					1.93

The $\frac{\beta_{1}}{\sum_{n=0}^{\infty }\beta_{n}}$ describes the relative importance of static economies of scale to the overall effect of volume.

The $\frac{\beta_{n}}{\beta_{n-1}}$ratio describes the learning-by-doing effect. A ratio > 1 would suggest incremental learning-by-doing. The absence of a consistent $\frac{\beta_{n}}{\beta_{n-1}}>1$ suggests some degradation of learning-by-doing.

### Sensitivity analysis

The first sensitivity analysis using the months as the time epoch for the learning-by-doing effect does not suggest a shorter time window for learning. The tests of joint significance for 1,2,3 and 4-month lags were p = 0.119, p = 0.019, p = 0.022 and p = 0.003 respectively. The second sensitivity analysis explored the model fit of the non-linear specification of volume. This analysis found the primary analysis to have a better model fit by information criteria (Table 4).

**Table 4:** Sensitivity analysis of the Probit regression model showing coefficients with standard errors, the proportion of the contemporaneous volume to the total volume effects and the ratio of lagged coefficients with volume specified as monthly volume and by the square root of quarterly volume.

Monthly sepsis volume	Models				
Lag depth	(1)	(2)	(3)	(4)	(5)
0	−0.00224***	−0.00166	−0.00104	−0.00078	−0.00030
	(0.0006)	(0.0007)	(0.0008)	(0.0008)	(0.0008)
1		−0.00166	−0.00058	−0.00028	−0.00012
		(0.0007)	(0.0006)	(0.00059)	(0.0006)
2			−0.00114*	−0.00092	−0.00067
			(0.0006)	(0.0006)	(0.0006)
3					−0.00023
					(0.0006)
4					−0.00151*
					(0.0006)
**Total**	−0.00224	−0.00255	−0.00276	−0.00259	−0.00282
$\frac{\beta_{1}}{\sum_{n=0}^{\infty }\beta_{n}}$	1.00	0.65	0.38	0.30	0.11
$\frac{\beta_{2}}{\beta_{3}}$			0.51	0.30	0.18
$\frac{\beta_{3}}{\beta_{4}}$				1.49	2.96
$\frac{\beta_{4}}{\beta_{5}}$					0.15
F statistic		2.68	6.33*	5.71*	9.83**
AIC	231787.8	227749.7	224164.6	220265.7	216676.2
BIC	232028.4	228000.4	224425.4	220536.4	216957
Square root of quarterly sepsis volume	Models				
**Lag depth**	**(1)**	**(2)**	**(3)**	**(4)**	**(5)**
0	−0.01622***	−0.00438	0.00657	0.00730	0.00240
	(0.0041)	(0.0055)	(0.0061)	(0.0055)	(0.0060)
1		−0.01178**	−0.00229	−0.00133	0.00090
		(0.0045)	(0.0050)	(0.0050)	(0.0053)
2			−0.02154***	−0.01544**	−0.01405**
			(0.0046)	(0.0051)	(0.0053)
3				−0.00953*	−0.00907
				(0.0047)	(0.0053)
4					0.00572
					(0.0051)
**Total**	−0.01622	−0.01612	−0.01726	−0.01900	−0.01410
$\frac{\beta_{1}}{\sum_{n=0}^{\infty }\beta_{n}}$	1.00	0.27	0.38	0.38	0.17
$\frac{\beta_{2}}{\beta_{3}}$			0.11	0.09	0.06
$\frac{\beta_{3}}{\beta_{4}}$				1.62	1.55
$\frac{\beta_{4}}{\beta_{5}}$					1.59
F statistic		6.96**	19.63***	20.84***	6.44*
AIC	231765.2	220499.7	210027.9	200350.7	188707.6
BIC	232005.8	220749.7	210287.2	200619.1	188984.8

P<0.05=*. P<0.01=**P<0.001=***.

The $\frac{\beta_{1}}{\sum_{n=0}^{\infty }\beta_{n}}$ describes the relative importance of static economies of scale to the overall effect of volume.

The $\frac{\beta_{n}}{\beta_{n-1}}$ratio describes the learning-by-doing effect. A ratio > 1 would suggest incremental learning-by-doing. The absence of a consistent $\frac{\beta_{n}}{\beta_{n-1}}>1$ suggests some degradation of learning-by-doing.

## Discussion

This study found a significant learning-by-doing effect for patients with sepsis treated in the ICU between 2010 and 2016. This study contrasts with previous studies by Gaynor et al and Ho et al which focused on cardiac procedures [9,13]. Learning-by-doing differs across conditions and experience may be more significant for sepsis than for elective cardiac procedures. Sepsis afflicts a wide spectrum of patients with varied comorbidities and requires a variety of interventions. This is unlike cardiac surgical patients who have similar risk factor profiles and require one of two procedure valve and coronary artery surgery. Outcomes for routine procedures would therefore likely depend on scale effects more than experience

In this study, accumulated experience is de not be completely retained between time periods, reflecting the cyclical nature of emergency medical teams. As doctors and nurses leave the service, the ICU loses the benefits of their accumulated experience [25]. Data from other healthcare contexts are consistent with the idea that the depreciation of experience is related to the staff turnover [26]. The inconsistent coefficients for the learning-by-doing effect with regards to sepsis might relate to the depreciation of organisational learning and the low temporal stability of ICU teams. The depreciation of knowledge for complex medical treatments has important implications for patient care. It is important that ICUs maintain caseload volumes over time to preserve institutional knowledge.

This study has limitations. Firstly, one of the challenges of including both contemporaneous and lagged volumes is the likely multicollinearity. ICUs that treat many sepsis patients in one quarter are likely to treat a large number in the following quarter. We use data from 231 ICUS and assume that the large sample size will contain sufficient variation between ICUs, which would weaken the collinearity between the contemporaneous and lagged volume. We undertook a robustness check that found a weak to moderate correlation between lagged volumes and low risk of multicollinearity. Secondly, this study does not contain details about compliance with evidence-based processes of care. Previous literature suggests higher volume hospitals have higher adherence to processes of care such as antibiotic administration and venous thrombo-embolism prophylaxis than lower-volume hospitals [27]. The adherence to processes of care also does not fully explain the volume-outcome relationship and we therefore contend that conclusions of this study would not be substantially altered by controlling for it [4,28].

## Conclusion and recommendations

This study finds that the underlying mechanism by which volume leads to improved outcomes is through learning-by-doing as opposed to the static scale effects of quality. ICUs improve by caring for a large volume of patients distributed over time, enabling continuous refinement of processes and skills. As populations age and medical care become increasingly complex, teams that can refine their skills over time will produce better outcomes. Centralization, while often proposed to enhance ICU quality, may fail to fully leverage the dynamic benefits of learning-by-doing and risks widening socioeconomic disparities in access to care [7,29]. The experience of the COVID-19 pandemic has highlighted how centralised models of care can struggle under the strain of unprecedented demand [30]. Considering the lessons from COVID-19, organizing ICUs to meet minimum volume standards without full centralization offers an optimal solution that supports equitable access while fostering continuous improvement. Policymakers must prioritize flexible and inclusive frameworks that prepare healthcare systems for future crises while maintaining high-quality critical care for all patients.

### Strengths and limitations of this study

- In terms of completeness, coverage and representativeness, this study includes 100% of all patients admitted to the ICU with sepsis in England Wales and Northern Ireland during the study period.
- A potential limitation is the collinearity of the contemporaneous and lagged volumes seen in other studies. This study addresses this by having a large sample size with sufficient variation between ICUs which weakens the collinearity between the contemporaneous and lagged volume.
- This study did not measure compliance with evidence-based processes of care. This unmeasured confounding could have a potential impact on the findings.
- Whilst the volume-outcome relationship has been described for a range of surgical conditions, less is known about its underlying mechanism. potential explanations are clinical experience and static economies scale.
- In this multicentre cohort study of 273001 patients across 231 ICUs with sepsis in the UK, the dynamic learning-by-doing effect was found to be more important than the static economies of scale effect.
- This finding suggests that ICUs caring for patients with sepsis, experience improved outcomes when caring for patients with sepsis. This argues against centralisation beyond meeting minimum volume standards.

## Supporting information

S1 Table Descriptive statistics. Trends in monthly, quarterly and six-monthly volumes from 2010 to 2016. Mean lagged volumes, cumulative volumes and difference in volumes.
(DOCX)

S1 Fig Correlation between successive lags described by violin plots (A) Volume qVolume q-1, (B) Volume q-1Volume q-2, (C) Volume q-2Volume q-3, and (D) Volume q-3Volume q-4. (DOCX)

## References

[1] Birkmeyer JD, Siewers AE, Finlayson EVA, Stukel TA, Lucas FL, Batista I, et al. Hospital volume and surgical mortality in the United States. N Engl J Med. 2002;346(15):1128–37. doi: 10.1056/NEJMsa012337 11948273

[2] Finks JF, Osborne NH, Birkmeyer JD. Trends in hospital volume and operative mortality for high-risk surgery. N Engl J Med. 2011;364(22):2128–37. doi: 10.1056/NEJMsa1010705 21631325 PMC3150488

[3] Maharaj R, McGuire A, Street A. Association of annual intensive care unit sepsis caseload with hospital mortality from sepsis in the United Kingdom, 2010-2016. JAMA Netw Open. 2021;4(6):e2115305. doi: 10.1001/jamanetworkopen.2021.15305 34185067 PMC8243236

[4] Mesman R, Westert GP, Berden BJMM, Faber MJ. Why do high-volume hospitals achieve better outcomes? A systematic review about intermediate factors in volume-outcome relationships. Health Policy. 2015;119(8):1055–67. doi: 10.1016/j.healthpol.2015.04.005 25958187

[5] Mesman R, Faber MJ, Berden BJJM, Westert GP. Evaluation of minimum volume standards for surgery in the Netherlands (2003-2017): A successful policy? Health Policy. 2017;121(12):1263–73. doi: 10.1016/j.healthpol.2017.09.017 29056240

[6] Nicholas LH, Stith SS. Are transplant centers that meet insurer minimum volume requirements better quality?. Med Care Res Rev. 2021;78(5):502–10. doi: 10.1177/1077558720919277 32418473

[7] Leung S, Pastores SM, Oropello JM, Lilly CM, Galvagno SM Jr, Badjatia N, et al. Regionalization of critical care in the United States: Current state and proposed framework from the academic leaders in critical care medicine task force of the society of critical care medicine. Crit Care Med. 2022;50(1):37–49. doi: 10.1097/CCM.0000000000005147 34259453

[8] Gaffney AW. Intensive care unit equity and regionalization in the COVID-19 era. Ann Am Thorac Soc. 2022;19(5):717–9. doi: 10.1513/AnnalsATS.202110-1200VP 35119976 PMC9116334

[9] Gaynor M, Seider H, Vogt WB. The volume–outcome effect, scale economies, and learning-by-doing. Am Econ Rev. 2005;95(2):243–7. doi: 10.1257/000282805774670329

[10] Van Gestel R, Müller T, Bosmans J. Does my high blood pressure improve your survival? Overall and subgroup learning curves in health. Health Econ. 2017;26(9):1094–109. doi: 10.1002/hec.3505 28449316

[11] Arrow KJ. The economic implications of learning by doing. Rev Econ Stud. 1962;29(3):155. doi: 10.2307/2295952

[12] Woods JR, Saywell RM Jr, Nyhuis AW, Jay SJ, Lohrman RG, Halbrook HG. The learning curve and the cost of heart transplantation. Health Serv Res. 1992;27(2):219–38. 1592606 PMC1069874

[13] Ho V. Learning and the evolution of medical technologies: The diffusion of coronary angioplasty. J Health Econ. 2002;21(5):873–85. doi: 10.1016/s0167-6296(02)00057-7 12349886

[14] Sfekas A. Learning, forgetting, and hospital quality: An empirical analysis of cardiac procedures in Maryland and Arizona. Health Econ. 2009;18(6):697–711. doi: 10.1002/hec.1400 18702083

[15] Ho SSY, Chan YL, Yeung DKW, Metreweli C. Blood flow volume quantification of cerebral ischemia: Comparison of three noninvasive imaging techniques of carotid and vertebral arteries. AJR Am J Roentgenol. 2002;178(3):551–6. doi: 10.2214/ajr.178.3.1780551 11856672

[16] Avdic D, Lundborg P, Vikström J. Estimating returns to hospital volume: Evidence from advanced cancer surgery. J Health Econ. 2019;63:81–99. doi: 10.1016/j.jhealeco.2018.10.005 30529091

[17] Ho V. Learning by doing. In: Culyer AJ, editor. Encyclopedia of Health Economics. San Diego: Elsevier; 2014. p. 141–5.

[18] Rudd KE, Johnson SC, Agesa KM, Shackelford KA, Tsoi D, Kievlan DR, et al. Global, regional, and national sepsis incidence and mortality, 1990-2017: Analysis for the Global Burden of Disease Study. Lancet. 2020;395(10219):200–11. doi: 10.1016/S0140-6736(19)32989-7 31954465 PMC6970225

[19] World Health A. Improving the prevention, diagnosis and clinical management of sepsis. Geneva: World Health Organization. 2017.

[20] Walton NT, Mohr NM. Concept review of regionalized systems of acute care: Is regionalization the next frontier in sepsis care?. J Am Coll Emerg Physicians Open. 2022;3(1):e12631. doi: 10.1002/emp2.12631 35024689 PMC8733842

[21] Young JD, Goldfrad C, Rowan K. Development and testing of a hierarchical method to code the reason for admission to intensive care units: The ICNARC coding method. Intensive Care National Audit & Research Centre. Br J Anaesth. 2001;87(4):543–8. doi: 10.1093/bja/87.4.543 11878722

[22] Harrison DA, Brady AR, Rowan K. Case mix, outcome and length of stay for admissions to adult, general critical care units in England, Wales and Northern Ireland: The intensive care national audit & research centre case mix programme database. Crit Care. 2004;8(2):R99-111. doi: 10.1186/cc2834 15025784 PMC420043

[23] Vandenbroucke JP, von Elm E, Altman DG, Gøtzsche PC, Mulrow CD, Pocock SJ, et al. Strengthening the reporting of observational studies in epidemiology (STROBE): Explanation and elaboration. Int J Surg. 2014;12(12):1500–24. doi: 10.1016/j.ijsu.2014.07.014 25046751

[24] Benkard CL. Learning and forgetting: The dynamics of aircraft production. Am Econ Rev. 2000;90(4):1034–54. doi: 10.1257/aer.90.4.1034

[25] Ervin JN, Kahn JM, Cohen TR, Weingart LR. Teamwork in the intensive care unit. Am Psychol. 2018;73(4):468–77. doi: 10.1037/amp0000247 29792461 PMC6662208

[26] Gowrisankaran G, Olin JM, editors. Causality, learning and forgetting in surgery. 2005.

[27] Bozic KJ, Maselli J, Pekow PS, Lindenauer PK, Vail TP, Auerbach AD. The influence of procedure volumes and standardization of care on quality and efficiency in total joint replacement surgery. J Bone Joint Surg Am. 2010;92(16):2643–52. doi: 10.2106/JBJS.I.01477 21084575

[28] Guarino M, Perna B, Cesaro AE, Maritati M, Spampinato MD, Contini C, et al. 2023 Update on sepsis and septic shock in adult patients: Management in the emergency department. J Clin Med. 2023;12(9):3188. doi: 10.3390/jcm12093188 37176628 PMC10179263

[29] Huguet M. Centralization of care in high volume hospitals and inequalities in access to care. Soc Sci Med. 2020;260:113177. doi: 10.1016/j.socscimed.2020.113177 32712556

[30] Carbonell R, Urgelés S, Rodríguez A, Bodí M, Martín-Loeches I, Solé-Violán J, et al. Mortality comparison between the first and second/third waves among 3,795 critical COVID-19 patients with pneumonia admitted to the ICU: A multicentre retrospective cohort study. Lancet Reg Health Eur. 2021;11:100243. doi: 10.1016/j.lanepe.2021.100243 34751263 PMC8566166

