# Supplementary material for: Does experience matter? Understanding the mechanism of the volume-outcome relationship: Learning-by-doing or economies of scale
Source: PLoS One. 2025 Mar 26;20(3):e0318808. doi: 10.1371/journal.pone.0318808 (PMC11940693; doi:10.1371/journal.pone.0318808)
Supplement: S1 Table — Trends in monthly, quarterly and six-monthly volumes from 2010 to 2016. Mean lagged volumes, cumulative volumes and difference in volumes. (DOCX) [file pone.0318808.s001.docx]

# Supplementary Appendix -S1

Table of Contents

[Supplementary Appendix 1](#_Toc190308219)

[1. S1 Table. Descriptive statistics. Trends in monthly, quarterly and six-monthly volumes from 2010 to 2016. Mean lagged volumes, cumulative volumes and difference in volumes. 2](#_Toc190308220)

## S1 Table. Descriptive statistics. Trends in monthly, quarterly and six-monthly volumes from 2010 to 2016. Mean lagged volumes, cumulative volumes and difference in volumes.

| **Volume** | **Year** | | | | | | | |
| --- | --- | --- | --- | --- | --- | --- | --- | --- |
|  | **2010** | **2011** | **2012** | **2013** | **2014** | **2015** | **2016** | **Total** |
| Median [IQR] | 19.1  [14.6,25,3] | 19  [15.1,25.9] | 22.8  [15.4,7.2] | 22.7  [16.5,29.8] | 22.3  [16.9,30.4] | 23.3  [17.3,31.0] | 24.1  [18.1,33.1] | 21.7  [18.1,29.3] |
| Mean (SD) | 20.8(8.8) | 21.3(9.2) | 22.8(10.3) | 24.2(11.0) | 24.9(11.2) | 25.1(10.8) | 25.7(10.3) | 23.8(10.5) |
| Lagged volume  Median[IQR] | 17.8  [12.4,21.9] | 18.8  [14.0,26.3] | 19.8  [14.3,25,6] | 20.7  [15.1,28.1] | 21.2  [15.9,28.9] | 22.0  [16.8,29.9] | 23.0  [16.8,31.6] | 20.2  [15.1,27.7] |
| Lagged volume  Mean (SD) | 18.2  (8.2) | 20.5  (9.3) | 21.4  (10.0) | 22.0  (10.8) | 23.3  (10.9) | 24.2  (10.9) | 24.4  10.1) | 22.  4(10.3) |
| Cumulative volume 3m | 55.1  (24.5) | 61.7  (27.8) | 64.3  (30.2) | 69.3  (32.6) | 70.1  (32.8) | 72.7  (32.8) | 73.2  (30.3) | 67.3  (31.1) |
| Cumulative volume 6m Mean (SD) | 109.5  (49.2) | 121.7  (55.0) | 127.3  (59.6) | 137.5  (64.3) | 139.8  (65.6) | 144.7  (65.5) | 145.8  (60.6) | 133.8  (61.9) |
| Cumulative volume 9m  Mean (SD) | 163.7  (70.1) | 181.7  (81.1) | 189.3  (88.1) | 205.6  (95.6) | 208.2  (98.3) | 216.7  (98.8) | 218.8  (91.2) | 200.0  (92.3) |
| Cumulative volume 12m Mean (SD) | N/A | 241.2  (107) | 250.2  (115.6) | 272  (127.4) | 278.5  (131.8) | 288.2  (132.4) | 291.9  (121.8) | 272.4  (125.0) |
| Difference in monthly volume,  Mean (SD) | -0.2(1.0) | 0.4(0.8) | 0.1(1.0) | 0.2(1.2) | 0.2(0.9) | 0.0(0.9) | 0.2(0.9) | 0.1(1.00) |
