# Supplementary material for: Does experience matter? Understanding the mechanism of the volume-outcome relationship: Learning-by-doing or economies of scale
Source: PLoS One. 2025 Mar 26;20(3):e0318808. doi: 10.1371/journal.pone.0318808 (PMC11940693; doi:10.1371/journal.pone.0318808)
Supplement: S1 Fig — (DOCX) [file pone.0318808.s002.docx]

# Supplementary Appendix

Table of Contents

[Supplementary Appendix 1](#_Toc190308308)

[2. S2 Figure. Correlation between successive lags described by violin plots (A) $\boldsymbol{Volume} \boldsymbol{qVolume} \boldsymbol{q}\mathbf{-1}$, (B) $\boldsymbol{Volume} \boldsymbol{q}\mathbf{-1}\boldsymbol{Volume} \boldsymbol{q}\mathbf{-2}$, (C) $\boldsymbol{Volume} \boldsymbol{q}\mathbf{-2}\boldsymbol{Volume} \boldsymbol{q}\mathbf{-3}$, and (D) $\boldsymbol{Volume} \boldsymbol{q}\mathbf{-3}\boldsymbol{Volume} \boldsymbol{q}\mathbf{-4}$. 2](#_Toc190308309)

## 2. S2 Figure. Correlation between successive lags described by violin plots (A) $\frac{{Volume}_{q}}{{Volume}_{q-1}}$, (B) $\frac{{Volume}_{q-1}}{{Volume}_{q-2}}$, (C) $\frac{{Volume}_{q-2}}{{Volume}_{q-3}}$, and (D) $\frac{{Volume}_{q-3}}{{Volume}_{q-4}}$.

The distribution of correlation coefficients between successive quartiles provides some indication of the risks of collinearity. The median correlation coefficient between $V_{q}$ and $V_{q-1}$ was 0.255 [IQR 0.056-0.467], median correlation coefficient between $V_{q-1}$ and $V_{q-2}$ was 0.290 [IQR 0.070-0.474] , median correlation coefficient between $V_{q-2}$ and $V_{q-3}$ was 0.267 [IQR 0.091-0.482] and the median correlation coefficient between $V_{q-3}$ and $V_{q-4}$ was 0.260 [IQR 0.062-0.491]. These values suggest a weak to moderate correlation between lagged volumes and low risk of multicollinearity.
